# Construction and Validation of a Serum Albumin-to-Alkaline Phosphatase Ratio-Based Nomogram for Predicting Pathological Complete Response in Breast Cancer

**DOI:** 10.3389/fonc.2021.681905

**Published:** 2021-10-08

**Authors:** Fanli Qu, Zongyan Li, Shengqing Lai, XiaoFang Zhong, Xiaoyan Fu, Xiaojia Huang, Qian Li, Shengchun Liu, Haiyan Li

**Affiliations:** ^1^ Department of Breast Surgery, The Sixth Affiliated Hospital, Sun Yat-Sen University, Guangzhou, China; ^2^ Department of Endocrine and Breast Surgery, The First Affiliated Hospital of Chongqing Medical University, Chongqing, China

**Keywords:** breast cancer, albumin-to-alkaline phosphatase ratio, neoadjuvant chemotherapy, nomogram, pathological complete response

## Abstract

**Background:**

Breast cancer patients who achieve pathological complete response (pCR) after neoadjuvant chemotherapy (NAC) have favorable outcomes. Reliable predictors for pCR help to identify patients who will benefit most from NAC. The pretreatment serum albumin-to-alkaline phosphatase ratio (AAPR) has been shown to be a prognostic predictor in several malignancies, but its predictive value for pCR in breast cancer is still unknown. This study aims to investigate the predictive role of AAPR in breast cancer patients and develop an AAPR-based nomogram for pCR rate prediction.

**Methods:**

A total of 780 patients who received anthracycline and taxane-based NAC from January 2012 to March 2018 were retrospectively analyzed. Univariate and multivariate analyses were performed to assess the predictive value of AAPR and other clinicopathological factors. A nomogram was developed and calibrated based on multivariate logistic regression. A validation cohort of 234 patients was utilized to further validate the predictive performance of the model. The C-index, calibration plots and decision curve analysis (DCA) were used to evaluate the discrimination, calibration and clinical value of the model.

**Results:**

Patients with a lower AAPR (<0.583) had a significantly reduced pCR rate (OR 2.228, 95% CI 1.246-3.986, *p*=0.007). Tumor size, clinical nodal status, histological grade, PR, Ki67 and AAPR were identified as independent predictors and included in the final model. The nomogram was used as a graphical representation of the model. The nomogram had satisfactory calibration and discrimination in both the training cohort and validation cohort (the C-index was 0.792 in the training cohort and 0.790 in the validation cohort). Furthermore, DCA indicated a clinical net benefit from the nomogram.

**Conclusions:**

Pretreatment serum AAPR is a potentially valuable predictor for pCR in breast cancer patients who receive NAC. The AAPR-based nomogram is a noninvasive tool with favorable predictive accuracy for pCR, which helps to make individualized treatment strategy decisions.

## Introduction

Breast cancer is the most common female malignancy and is one of the leading causes of female cancer morbidity and mortality (1). Neoadjuvant chemotherapy (NAC) is a standard treatment option for most breast cancer patients, especially those with locally advanced or inoperable breast cancer. It aims to reduce disease burden and make breast cancer operable in locally advanced patients (2). For operable breast cancer, NAC makes it possible to receive breast-conserving surgery. Furthermore, NAC provides an opportunity for the assessment of chemosensitivity *in vivo* to guide individualized further systematic therapy based on tumor response (3). A recent meta-analysis of 52 clinical trials, including 27,895 patients, demonstrated that pathological complete response (pCR) after NAC was associated with better event-free survival and overall survival in all breast cancer molecular subtypes (4). However, the response to NAC varies among different molecular subtypes and histopathological characteristics (5). NAC might increase the risk of disease progression in chemoresistant tumors by delaying surgery. A portion of patients do not benefit from NAC and are exposed to the toxicity of the chemotherapy drugs unnecessarily. Thus, there is an urgent need to seek an effective method that can predict pCR for the identification of patients who will benefit most from NAC.

Previous studies demonstrated that various methods could be applied to predict pCR with NAC in breast cancer patients, including analysis of gene signatures, histomorphological factors and imaging features (6–8). Compared with these methods, serum samples are easy to obtain and reflect the comprehensive state of cancer patients. Various serum tumor markers have been identified as prognostic predictive factors in breast cancer patients, such as CEA, CA15-3, CA19-9 and CA125 (9, 10). It is known that systemic inflammation accelerates tumor progression (11). Inflammation-based prognostic scores, including the neutrophil to lymphocyte ratio, lymphocyte to monocyte ratio, platelet to lymphocyte ratio and C-reactive protein/albumin ratio, have been analyzed to evaluate their prognostic predictive value in breast cancer, but the results are controversial (12, 13). Albumin (ALB) is the most abundant serum protein and is synthesized by the liver. It can be regarded as a surrogate parameter for systemic inflammation because of its important function as an immunomodulatory molecule (14). Several studies have found that ALB is a reliable factor for predicting prognosis in different types of cancer, including gynecological cancers, gastrointestinal cancers, hepatocellular carcinoma and esophageal squamous cell carcinoma (15–18). Serum alkaline phosphatase (ALP) is derived from the liver, skeletal tissue, intestine, kidney, placenta and a variety of tumors and is conventionally regarded as a serum marker of hepatobiliary pathology and fracture (19). Previous studies have suggested that ALP is associated with systemic inflammation and tumor development (20, 21). The albumin-to-alkaline phosphatase ratio (AAPR) is a combined index associated with systemic inflammation, which is calculated by dividing the ALB level by the ALP level. It was first reported as a novel prognostic indicator in hepatocellular carcinoma (22). AAPR is based on low-cost routine blood test indexes and has a better prognostic predictive effect than ALB or ALP alone (23). Patients with higher AAPR have better survival outcomes than those with lower AAPR in various cancers, including breast cancer (24). However, whether AAPR can be used as a predictor for pCR in breast cancer patients receiving NAC is still unclear.

Nomograms, graphic illustrations based on regression models, are considered comprehensive predictive tools of patient outcomes. They have been widely used in cancer prognosis prediction models. Although several AAPR-based nomograms have been developed to predict survival in various cancer patients, nomograms based on AAPR predicting the probability of pCR in breast cancer patients are still scant. In our current study, a nomogram based on AAPR and clinicopathological variables was developed and validated to predict the individual probability of pCR in breast cancer patients who received NAC.

## Methods

### Study Population

We retrospectively reviewed the medical records of primary breast cancer patients at the First Affiliated Hospital of Chongqing Medical University from January 2012 to March 2018. The inclusion criteria were as follows: 1) pathological diagnosis of invasive breast cancer; 2) female; 3) received NAC and surgery; 4) received at least 3 courses of treatment with TEC (docetaxel 75 mg/m^2^, epirubicin 75 mg/m^2^ and cyclophosphamide 500 mg/m^2^ each 21 days before surgery; and 5) serum ALB and ALP levels were measured before treatment. Patients with prior history of malignancies or without complete information were excluded. Ultimately, a total of 780 patients were included for analysis. They were randomly divided into the training cohort and the validation cohort at a ratio of 7:3 (training group: n=546, validation group: n=234). This study was reviewed and approved by the ethics committee of the First Affiliated Hospital of Chongqing Medical University.

### Data Collection

Clinical characteristics collected for further analysis included age, menopausal status, courses of NAC, histological type of cancer, tumor size, clinical nodal status, histological grade, estrogen receptor (ER) status, progesterone receptor (PR) status, human epidermal growth factor receptor-2 (HER2) receptor status, Ki67 status, and serum ALB and ALP levels. The tumor size was assessed using ultrasonography by the specific ultrasound operators working in our Breast Cancer Center. ER and PR expression were defined as positive when greater than 1% of the tumor cells exhibited nuclear staining on immunohistochemistry. HER2 positivity was defined as 3+ by immunohistochemical staining or an over 2.0-fold increase by fluorescence *in situ* hybridization (25). The Ki67 value was defined as the percentage of Ki67-positive cells (500-1,000) among the total number of cancer cells in the invasive front of the tumor (26). ER, PR, HER2 and Ki67 were assessed by two pathologists independently. Tumors were classified into four categories based on the expression of ER, PR, and HER2: luminal subtype (ER+ and/or PR+, HER2-), luminal/HER2 subtype (ER+ and/or PR+, HER2+), HER2 enriched subtype (ER-, PR-, HER2+), and TNBC subtype (ER-, PR-, HER2-). ALB and ALP were tested along with routine plasma examinations at diagnosis. Blood samples were collected into coagulant-coated tubes after patients had fasted for at least 6 hours. ALB and ALP were analyzed by a fully automatic biochemical analyzer (Roche c701, Basel, Switzerland). Pathological complete response (pCR) was recognized as the absence of any residual tumor lesions in any excised breast tissue or lymph node according to the Miller-Payne grading system (27).

### Statistical Analysis

The cutoff values of ALB and ALP were 40 g/L and 100 U/L, respectively, which were established based on the normal reference value. The optimal cutoff value of AAPR was determined by the maximum Youden index through receiver operating characteristic (ROC) curve analysis. The chi-square test and Fisher’s exact test were used to evaluate the differences in clinicopathological variables between the training cohort and the validation cohort. In addition, the associations between AAPR and clinicopathological characteristics in breast cancer patients were assessed by the chi-square test or Fisher’s exact test. Similarly, the relationships between pCR and clinicopathological characteristics were analyzed. The primary goal of this study was to estimate the likelihood of breast cancer patients reaching pCR after NAC. Multivariate logistic regression analysis was performed to assess the associations between clinicopathological factors and the likelihood of pCR. Odds ratios were reported with corresponding 95% confidence intervals (CIs). An individualized nomogram was constructed based on the logistic regression model with the *rms* package in R software. The performance of the model was evaluated by discrimination and calibration in both the training cohort and the validation cohort. By testing the concordance between the prediction probability and the actual state, the concordance index (C-index) was calculated to assess the prediction and discrimination ability of the model. Calibration of the nomogram assessed by internal validation through 1000 bootstrap resamples was shown by a calibration curve. The fitness of the model was analyzed by the Hosmer-Lemeshow test. Furthermore, decision curve analysis was applied to assess the net benefit of the nomogram.

All statistical analyses were performed by using the Statistical Package for the Social Sciences version 25.0 software (IBM Corp., Armonk, USA) and R software (version 4.0.3; https://www.R-project.org/). A two-sided *p*-value < 0.05 was considered statistically significant.

## Results

### Patient Characteristics

A total of 780 eligible patients were enrolled in this study according to the inclusion criteria. They were randomly divided into the training cohort and the validation cohort at a ratio of 7:3 to develop and validate the predictive model. The mean age of all patients at baseline was 49.0 ± 9.1 years (range 20.0-72.0 years), and 60.0% (n=468) of them were premenopausal. Most of the patients (n=749, 96.0%) were diagnosed with invasive lobular carcinoma. The mean tumor size was 4.0 ± 2.1 cm at baseline, while it was reduced to 2.1 ± 1.7 cm after NAC. Moreover, 461 (59.1%) patients had node-positive disease at baseline. The proportions of ER-positive, PR-positive, HER2-positive patients were 62.8% (n=490), 48.8% (n=381) and 41.8% (n=326), respectively, among 780 patients. More than half of the patients (n=539, 69.1%) had Ki67 expression ≥ 14%. In addition, 90.6% (n=707) of patients had normal serum ALP levels, while 60.1% (n=469) of patients had normal serum ALB levels. According to the Miller-Payne grading system, 103 (13.2%) patients were evaluated as having pCR after NAC. As shown in **Table 1**, no significant difference was observed in clinicopathological factors between the training cohort and the validation cohort.

**Table 1:** Clinicopathological characteristics in the training, validation and overall cohorts.

Characteristics	Overall (n = 780)	Training cohort (n = 546)	Validation cohort (n = 234)	*p*-value
**Age (y)**				0.672
<50	429 (55.0)	303 (70.1)	126 (53.8)	
≥50	351 (45.0)	243 (29.9)	108 (46.2)	
**Menopause**				0.307
Yes	312 (40.0)	212 (38.8)	100 (42.7)	
No	468 (60.0)	334 (61.2)	134 (57.3)	
**Chemotherapy cycles**				0.523
3	22 (2.8)	17 (3.1)	5 (2.1)	
4	695 (89.1)	488 (89.4)	207 (88.5)	
5-8	63 (8.1)	41 (7.5)	22 (9.4)	
**Histological type**				0.902
Ductal	749 (96.0)	523 (95.8)	226 (96.6)	
Lobular	10 (1.3)	8 (1.5)	2 (0.9)	
Others	21 (2.7)	15 (2.7)	6 (2.6)	
**Tumor size**				0.610
T1	83 (10.6)	62 (11.4)	21 (9.0)	
T2	538 (69.0)	373 (68.3)	165 (70.5)	
T3	159 (20.4)	111 (20.3)	48 (20.5)	
**Clinical nodal status**				0.063
Negative	319 (40.9)	235 (43.0)	84 (35.9)	
Positive	461(59.1)	311 (57.0)	150 (64.1)	
**Histological Grade**				0.397
I	50 (6.4)	37 (6.8)	13 (5.6)	
II	575 (73.7)	407 (74.5)	168 (71.8)	
III	155 (19.9)	102 (18.7)	53 (22.6)	
**ER**				0.628
Negative	290 (37.2)	206 (37.7)	84 (35.9)	
Positive	490 (62.8)	340 (62.3)	150 (64.1)	
**PR**				0.913
Negative	399 (51.2)	280 (51.3)	119 (50.9)	
Positive	381 (48.8)	266 (48.7)	115 (49.1)	
**HER2 status**				0.163
Negative	454 (58.2)	309 (56.6)	145 (62.0)	
Positive	326 (41.8)	237 (43.4)	89 (38.0)	
**Ki67 expression (%)**				
<14	241 (30.9)	167 (30.6)	74 (31.6)	0.774
≥14	539 (69.1)	379 (69.4)	160 (68.4)	
**Molecular subtypes**				0.277
Luminal	332 (42.6)	230 (42.1)	102 (43.6)	
Luminal/HER2	175 (22.4)	123 (22.5)	52 (22.2)	
HER2	151 (19.4)	114 (20.9)	37 (15.8)	
TNBC	122 (15.6)	79 (14.5)	43 (18.4)	
**ALB**				0.363
<40	311 (39.9)	212 (38.8)	99 (42.3)	
≥40	469 (60.1)	334 (61.2)	135 (57.7)	
**ALP**				0.406
<100	707 (90.6)	498 (91.2)	209 (89.3)	
≥100	73 (9.4)	48 (8.8)	25 (10.7)	
**AAPR**				0.498
<0.583	339 (43.5)	233 (42.7)	106 (45.3)	
≥0.583	441 (56.5)	313 (57.3)	128 (54.7)	
**Response evaluation**				0.661
pCR	103 (13.2)	74 (13.6)	29 (12.4)	
Non-pCR	677 (86.8)	472 (86.4)	205 (87.6)	

ER, estrogen receptor; PR, progesterone receptor; HER2, human epidermal growth factor 2; ALB, albumin; ALP, alkaline phosphatase; AAPR, albumin-to-alkaline phosphatase ratio; pCR, pathologic complete response.

### Associations Between AAPR and Clinicopathological Characteristics

The relationships between AAPR and clinicopathological characteristics in the training cohort were assessed (**Table 2**). The optimal cutoff value of AAPR was 0.583, according to the ROC curve. Following the cutoff value, 233 (42.7%) patients were included in the high-AAPR group (AAPR<0.583), while the other 313 (57.3%) patients were included in the low-AAPR group (AAPR≥0.583). The results revealed that AAPR level was significantly associated with age (*p*<0.001), menopausal status (*p*<0.001), histological type (*p*=0.007), molecular subtypes (*p*=0.045) and pCR (*p*=0.030). No differences were observed in chemotherapy cycles, tumor size, clinical nodal status, histological grade, ER, PR, HER2 or Ki67 between the two groups.

**Table 2:** Correlations between AAPR and clinicopathological characteristics in the training cohort.

Characteristics	AAPR < 0.583 (n = 233)	AAPR ≥ 0.583 (n = 313)	*p*-value
**Age (y)**			<0.001
<50	82 (35.2)	221 (70.6)	
≥50	151 (64.8)	92 (29.4)	
**Menopause**			<0.001
Yes	133 (57.1)	79 (25.2)	
No	100 (42.9)	234 (74.8)	
**Chemotherapy cycles**			0.924
3	8 (3.4)	9 (2.9)	
4	208 (89.3)	280 (89.5)	
5-8	17 (7.3)	24 (7.7)	
**Histological type**			0.007
Ductal	219 (94.0)	304 (97.1)	
Lobular	2 (0.9)	6 (1.9)	
Others	12 (5.2)	3 (1.0)	
**Tumor size**			0.188
T1	23 (9.9)	39 (12.5)	
T2	169 (72.5)	204 (65.2)	
T3	41 (17.6)	70 (22.4)	
**Clinical nodal status**			0.960
Negative	100 (42.9)	135 (43.1)	
Positive	133 (57.1)	178 (56.9)	
**Histological Grade**			0.189
I	21 (9.0)	16 (5.1)	
II	171 (73.4)	236 (75.4)	
III	41 (17.6)	61 (19.5)	
**ER**			0.277
Negative	94 (40.3)	112 (35.8)	
Positive	139 (59.7)	201 (64.2)	
**PR**			0.260
Negative	126 (54.1)	154 (49.2)	
Positive	107 (45.9)	159 (50.8)	
**HER2 status**			0.396
Negative	127 (54.5)	182 (58.1)	
Positive	106 (45.5)	131 (41.9)	
**Ki67 expression (%)**			0.608
<14	74 (31.8)	93 (29.7)	
≥14	159 (68.2)	220 (70.3)	
**Molecular subtypes**			0.045
Luminal	97 (41.6)	133 (42.5)	
Luminal/HER2	45 (19.3)	78 (24.9)	
HER2	61 (26.2)	53 (16.9)	
TNBC	30 (12.9)	49 (15.7)	
**Response evaluation**			0.030
pCR	23 (9.9)	51 (16.3)	
Non-pCR	210 (90.1)	262 (83.7)	

ER, estrogen receptor; PR, progesterone receptor; HER2, human epidermal growth factor 2; AAPR, albumin-to-alkaline phosphatase ratio; pCR, pathologic complete response.

### Predictors of pCR

In univariate analysis of the training cohort (**Table 3**), pCR was significantly correlated with tumor size, clinical nodal status, histological grade, ER, PR, Ki67, molecular subtypes and AAPR. Multivariate logistic regression models were applied to adjust for potential confounders. Variables with p < 0.05 in univariate analysis were included in multivariable models. To avoid the influence of multicollinearity between ER and molecular subtypes, only one of them was applied to the final model. Tumor size, clinical nodal status, histological grade, PR, Ki67 and AAPR were indicated as independent predictors for pCR in breast cancer patients who received NAC. Compared with patients with lower AAPR (AAPR<0.583), the probability of pCR in those with higher AAPR was 2.228-fold higher (95% CI 1.246-3.986, *p*=0.007). Patients with larger and higher historical grade tumors were less likely to achieve pCR (adjusted OR 0.355, 95% CI 0.168-0.752, *p*=0.007 for T2; adjusted OR 0.237, 95% CI 0.091-0.620, *p*=0.003 for T3; adjusted OR 0.256, 95% CI 0.105-0.624, *p*=0.003 for Grade II; adjusted OR 0.247, 95% CI 0.087-0.702, *p*=0.009 for Grade III) (**Table 4**). Patients with node-positive and PR-positive diseases had more difficulty achieving pCR than those with node-negative and PR-negative diseases (adjusted OR 0.288, 95% CI 0.162-0.513, *p*<0.001 for node-negative status; adjusted OR 0.462, 95% CI 0.220-0.971, *p*=0.041 for PR-negative status). Moreover, the probability of pCR in patients with higher Ki67 levels was 3.334-fold (95% CI 1.534-7.229, *p*=0.002) higher than that in patients with lower Ki67 levels.

**Table 3:** Univariate analysis for factors associated with pCR in the training cohort.

Characteristics	Non-pCR (n = 472)	pCR (n = 74)	*p*-value
**Age (y)**			0.306
<50	266 (56.4)	37 (50.0)	
≥50	206 (43.6)	37 (50.0)	
**Menopause**			0.561
Yes	181 (38.3)	31 (41.9)	
No	291 (61.7)	43 (58.1)	
**Chemotherapy cycles**			0.947
3	15 (3.2)	2 (2.7)	
4	422 (89.4)	66 (89.2)	
5-8	35 (7.4)	6 (8.1)	
**Histological type**			0.609
Ductal	453 (96.0)	70 (94.6)	
Lobular	7 (1.5)	1 (1.4)	
Others	12 (2.5)	3 (4.1)	
**Tumor size**			0.007
T1	46 (9.7)	16 (21.6)	
T2	325 (68.9)	48 (64.9)	
T3	101 (21.4)	10 (13.5)	
**Clinical nodal status**			<0.001
Negative	181 (38.3)	54 (73.0)	
Positive	291 (61.7)	20 (27.0)	
**Histological Grade**			0.011
I	26 (5.5)	11 (14.9)	
II	358 (75.8)	49 (66.2)	
III	88 (18.6)	14 (18.9)	
**ER**			<0.001
Negative	164 (34.7)	42 (56.8)	
Positive	308 (65.3)	32 (43.2)	
**PR**			<0.001
Negative	226 (47.9)	54 (73.0)	
Positive	246 (52.1)	20 (27.0)	
**HER2 status**			0.431
Negative	264 (55.9)	29 (39.2)	
Positive	208 (44.1)	45 (60.8)	
**Ki67 expression (%)**			<0.001
<14	158 (33.5)	9 (12.2)	
≥14	314 (66.5)	65 (87.8)	
**Molecular subtypes**			<0.001
Luminal	210 (44.5)	20 (27.0)	
Luminal/HER2	109 (23.1)	14 (18.9)	
HER2	99 (21.0)	15 (20.3)	
TNBC	54 (11.4)	25 (33.8)	
**ALB**			0.765
<40	178 (37.7)	34 (45.9)	
≥40	294 (62.3)	40 (54.1)	
**ALP**			0.823
<100	430 (91.1)	68 (91.9)	
≥100	42 (8.9)	6 (8.1)	
**AAPR**			0.030
<0.583	210 (44.5)	23 (31.1)	
≥0.583	262 (55.5)	51 (68.9)	

ER, estrogen receptor; PR, progesterone receptor; HER2, human epidermal growth factor 2; ALB, albumin; ALP, alkaline phosphatase; AAPR, albumin-to-alkaline phosphatase ratio; pCR, pathologic complete response.

**Table 4:** Multivariate analysis for factors associated with pCR in the training cohort.

Characteristics	Crude OR (95%CI)	*p*-value	Adjusted OR (95%CI)	*p*-value
**Tumor size**				
T1	Reference		Reference	
T2	0.425 (0.233-0.809)	0.009	0.355 (0.168-0.752)	0.007
T3	0.285 (0.120-0.675)	0.004	0.237 (0.091-0.620)	0.003
**Clinical nodal status**				
Negative	Reference		Reference	
Positive	0.230 (0.134-0.398)	<0.001	0.288 (0.162-0.513)	<0.001
**Histological grade**				
I	Reference		Reference	
II	0.324 (0.150-0.696)	0.004	0.256 (0.105-0.624)	0.003
III	0.376 (0.152-0.927)	0.034	0.247 (0.087-0.702)	0.009
**ER**				
Negative	Reference		Reference	
Positive	0.406 (0.247-0.667)	<0.001	0.789 (0.393-1.584)	0.505
**PR**				
Negative	Reference		Reference	
Positive	0.340 (0.198-0.586)	<0.001	0.462 (0.220-0.971)	0.041
**Ki67 expression (%)**				
<14	Reference		Reference	
≥14	3.634 (1.764-7.487)	<0.001	3.334 (1.534-7.229)	0.002
**AAPR**				
<0.583	Reference		Reference	
≥0.583	1.777 (1.052-3.004)	0.032	2.228 (1.246-3.986)	0.007

ER, estrogen receptor; PR, progesterone receptor; AAPR, albumin-to-alkaline phosphatase ratio; pCR, pathologic complete response.

Considering the high heterogeneity of breast cancer, univariate and multivariate analyses were performed in different subtypes. As shown in **Supplementary Tables 1, 2**, tumor size, histological grade, Ki67 and AAPR were indicated as independent predictors for pCR in the luminal subtype. In the luminal/HER2 subtype, the independent predictors were clinical nodal status, histological grade and Ki67 (**Supplementary Tables 3, 4**). In the HER2 enriched subtype, only clinical nodal status and histological grade were statistically significant in multivariate analysis (**Supplementary Tables 4, 5**). In the TNBC subtype, tumor size, clinical nodal status and AAPR were identified as independent predictors for pCR (**Supplementary Tables 7, 8**). The results demonstrated that the probability of pCR in patients with higher AAPR was 3.245-fold higher (95% CI 1.055-9.980, *p*=0.040) in the luminal subtype and 2.868-fold higher (95% CI 1.048-7.849, *p*=0.040) in the TNBC subtype when compared with those with lower AAPR (AAPR<0.583). It is consistent with the results obtained in the overall analysis. However, AAPR was not correlated with the pCR rate in the luminal/HER2 (*p*=0.215) and the HER2 enriched (*p*=0.853) subtype.

### Development and Validation of the Nomogram

**Figure 1:**
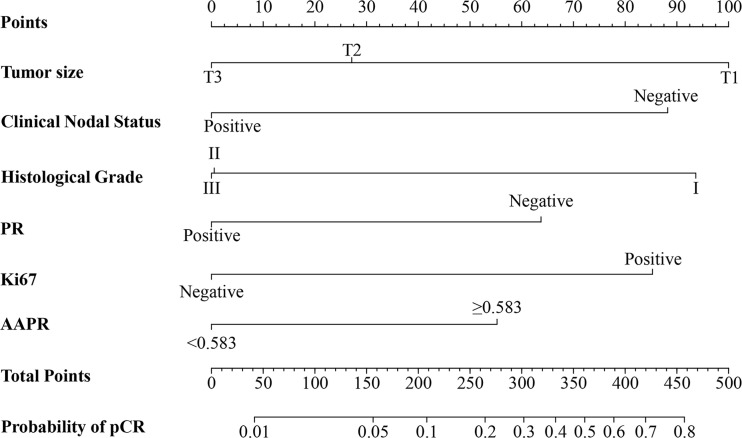
The AAPR-based nomogram for predicting the probability of pCR after NAC in breast cancer patients. PR, progesterone receptor; AAPR, albumin-to-alkaline phosphatase ratio; pCR, pathologic complete response.

A nomogram was constructed based on the multivariate regression analysis of the training cohort. In **Figure 1**, tumor size, clinical nodal status, histological grade, PR, Ki67 and AAPR were used to calculate points based on the points scale axis. The sum of all these points provides a total point to estimate the probability of pCR.

**Figure 2:**
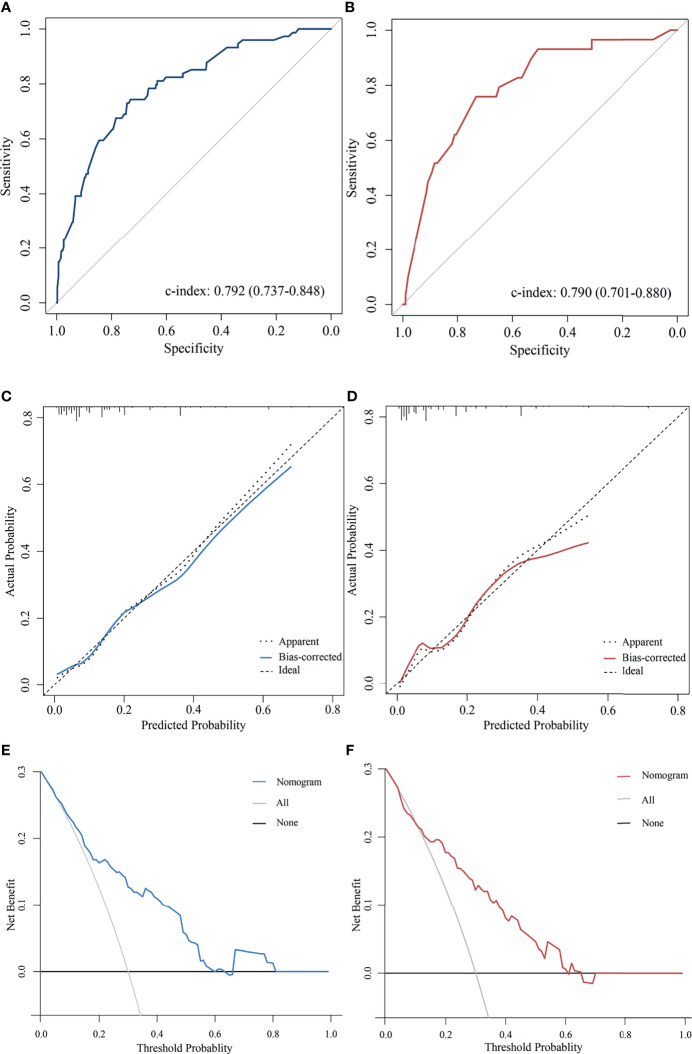
Validation the predictive value of the AAPR-based nomogram. The ROC curves for the nomogram model in **(A)** the training cohort and **(B)** validation cohort. The calibration plots for the nomogram model in **(C)** the training cohort and **(D)** validation cohort. The decision curves show the net-benefit of using the nomogram in **(E)** the training cohort and **(F)** validation cohort.

The predictive accuracy of the nomogram for the pCR rate of breast cancer patients who received NAC measured by the C-index was 0.792 (95% CI 0.737-0.848) in the training cohort and 0.790 (95% CI 0.701-0.880) in the validation cohort (**Figures 2A, B** **)**. In addition, the calibration curves for pCR demonstrated a satisfactory fit between the prediction and the actual values (**Figures 2C, D** **)**. As shown in **Figures 2E, F**, decision curves were illustrated for the constructed nomogram. It suggested that, for predicted probability thresholds between 0 and 60% the model-based decision was superior to either the treat-none or the treat-all-patients scheme.

## Discussion

NAC was first introduced in the 1970s (28). It is now widely used among breast cancer patients. Patients attaining pCR after NAC have better survival outcomes regardless of molecular subtype (4); however, the tumor response to NAC varies. Therefore, an accurate prediction assessment for pCR after NAC in breast cancer patients has great clinical significance. In the present study, the predictive value of AAPR for the probability of pCR was analyzed in breast cancer patients who received NAC. The results demonstrate that AAPR is an independent predictive factor. Pretreatment AAPR under 0.583 is associated with a lower pCR rate. More importantly, a novel AAPR-based nomogram was constructed to quantify the probability of pCR, which has promising prospects for clinical use.

In recent years, an accumulating body of research has found that serum parameters can be utilized as predictive factors in breast cancer, such as serum fibrinogen, D-dimer, lipid profiles, lymphocyte to monocyte ratio and platelet to lymphocyte ratio (12, 29). ALB and ALP are two accessible routine laboratory indexes. ALB is a globular, water-soluble protein that is exclusively produced and secreted by hepatocytes. Since ALB accounts for approximately half of the total serum protein, it is the most abundant protein in serum (14). Previous studies have confirmed that hypoalbuminemia is associated with inflammation and malnutrition during cancer development and progression (30). This may be explained by reduced synthesis, increased consumption and loss of serum ALB (31). ALB also contributes to balancing cell proliferation and metabolism (32). Hypoalbuminemia may reflect impairment of immunity and affect the response to anticancer treatment (33). Several studies have demonstrated that pretreatment ALB is a prognostic factor in various cancers, including lung, pancreatic, gastric, and colorectal cancers (34). ALP is a hydrolytic enzyme that dephosphorylates different types of molecules, including nucleotides, proteins, and alkaloids (35). It plays an anti-inflammatory and tissue-protective role by enhancing the conversion of ATP into adenosine and increasing the level of adenosine (36). A previous study demonstrated that the activity of ALP is associated with cancer cell death, migration and mesenchymal-to-epithelial transition (21). It was reported that a heavy tumor burden and tumor metastasis might result in the elevation of ALP. Higher serum ALP levels were demonstrated to be related to a worse prognosis in nasopharyngeal carcinoma, prostate cancer and colorectal cancer (37–39). Chen et al. (40) reported that the pretreatment serum ALP level was an independent unfavorable prognostic factor for disease-free survival and overall survival in TNBC patients. AAPR is a novel, accessible, low-cost and noninvasive index that is calculated based on ALB and ALP and potentially reflects systematic inflammation and nutrition status. It was first reported as a prognostic factor in hepatocellular carcinoma and has already been identified as a prognostic predictor in various cancers (22). Kim et al. (33) reported that a high AAPR was related to better overall survival, progression-free survival, and locoregional relapse-free survival in nasopharyngeal carcinoma. Li et al. (23) also reported that AAPR was an independent predictive factor for PFS in small-cell lung cancer. A retrospective study enrolled 746 nonmetastatic breast cancer patients and suggested that a lower AAPR was related to shorter OS (41).

The results of these previous studies suggested that a high AAPR was associated with better survival outcomes. However, the predictive value of AAPR for pCR possibility in breast cancer patients who received NAC remains unknown. In the present study, the optimal cutoff value of AAPR was 0.583 with the maximum Youden index. Initially, we evaluated the relationship between AAPR and breast cancer characteristics, and our results suggested that AAPR was significantly associated with age (*p*<0.001), menopausal status (*p*<0.001), histological type (*p*=0.007), molecular subtypes (*p*=0.045) and pCR (*p*=0.030). Our further analysis focused on assessing the predictive value of clinicopathological factors. We found that the pretreatment ALB and ALP levels of most patients were within the normal range. Moreover, the univariate analysis indicated that neither pretreatment ALB nor ALP could be used as a predictor for pCR in breast cancer patients. Nevertheless, AAPR is an independent predictive factor, and patients with low AAPR had a significantly lower probability of achieving pCR.

Multivariate analysis also suggested that tumor size, clinical nodal status, histological grade, PR and Ki67 expression were independent predictive factors. A retrospective study by Briete et al. (n= 2366) demonstrated that lower T-stages had significantly higher pCR rates than higher T-stages (42), and a study by Bonadonna et al. (*n* = 165) showed that the tumor response was inversely proportional to initial tumor size for tumors larger than 3 cm (43). These results emphasized the importance to consider tumor size when estimating the chance of pCR in breast cancer patients. The CTNeoBC pooled analysis included 11955 patients suggested that the frequency of pCR in patients with clinical-nodal-positive and hormone-receptor-positive tumors was low (44). Ki67 expression was related to tumor cell proliferation, several studies revealed that patients with higher Ki67 expression were more likely to achieve pCR (45–47).Most of the above indicators are consistent with the existing literature. However, there was no significant correlation between HER2 status and pCR, which is inconsistent with previous studies (4). Moreover, the overall pCR rate in the current study was 13.2%, which is lower than that in some previous large-scale studies (20.4-21.1%) (4, 48). The NOAH trial and the NeoSphere trial demonstrated the addition of neoadjuvant trastuzumab and pertuzumab to neoadjuvant chemotherapy significantly improved the pCR rate in the HER2-positive disease (49, 50). However, 97% of HER2-positive patients refused anti-HER2 therapy in our study owing to the high costs. The absence of neoadjuvant anti-HER2 therapy has a great impact on the pCR rate of HER2-positive patients, which may result in the relatively lower pCR rate and the insignificant predictive value of AAPR in the luminal/HER2 and the HER2 enriched subtypes. This phenomenon also confirmed the importance of HER2-targeted therapy during NAC in HER2-positive patients. To the best of our knowledge, this is the first retrospective study conducted to analyze the predictive value of AAPR for pCR in breast cancer patients who received NAC. Based on the above results, a serum AAPR-based nomogram was developed and validated to quantitatively estimate the pCR probability in patients who received NAC.

However, there are several limitations. First, this study was a retrospective study conducted at a single center. The training cohort and validation cohort minimize the possible selection bias, but the optimal cutoff value of AAPR and nomogram require further external validation. In addition, only 3% of HER2-positive patients received trastuzumab therapy during NAC due to financial issues, which may have affected the pCR rate and the predictive role of AAPR in the HER2-positive patients. Third, although this study included clinicopathological information as comprehensively as possible from medical records, some valuable factors may still exist that were not available in our analysis. It is necessary to further analyze the predictive role of AAPR in the HER2-positive subgroup with adequately treated patients in the future. Larger multicenter prospective clinical studies are needed to improve and validate the AAPR-based nomogram in breast cancer patients with different molecular subtypes and treated with more innovative therapeutic modalities. Laboratory experiments are required to explore the mechanism of the predictive capability of AAPR for pCR in breast cancer patients.

## Conclusion

In conclusion, the present study demonstrated that pretreatment serum AAPR, tumor size, clinical nodal status, histological grade, PR and Ki67 expression were independent predictive factors for pCR in breast cancer patients treated with NAC. The AAPR-based nomogram can accurately estimate pCR probability and helps to determine individual treatment strategies.

## Data Availability Statement

The datasets used and analyzed during the current study are available from the corresponding author on reasonable request.

## Ethics Statement

This study was reviewed and approved by the ethics committee of the First Affiliated Hospital of Chongqing Medical University. Written informed consent was obtained from all patients included in the study.

## Author Contributions

FQ conceived the study, conducted most of the data analysis and drafted the manuscript. ZL, SQL, and XZ participated in the data analysis. XF, XH, and QL participated in the figure production. SCL provided the original data and made detailed revisions to the manuscript. HL guided the entire analysis process and determined the direction of the research for each section. All authors contributed to the article and approved the submitted version.

## Funding

This work was supported by the Science and Technology Planning Project of Guangzhou (No. 805275295029), the Natural Science Foundation of Guangdong Province (No.2020A1515010425), and the China Postdoctoral Science Foundation (No. 2020M672986, 2020M670108ZX).

## Conflict of Interest

The authors declare that the research was conducted in the absence of any commercial or financial relationships that could be construed as a potential conflict of interest.

## Publisher’s Note

All claims expressed in this article are solely those of the authors and do not necessarily represent those of their affiliated organizations, or those of the publisher, the editors and the reviewers. Any product that may be evaluated in this article, or claim that may be made by its manufacturer, is not guaranteed or endorsed by the publisher.
