# Supplementary material for: Construction and Validation of a Serum Albumin-to-Alkaline Phosphatase Ratio-Based Nomogram for Predicting Pathological Complete Response in Breast Cancer
Source: Front Oncol. 2021 Oct 8;11:681905. doi: 10.3389/fonc.2021.681905 (PMC8531528; doi:10.3389/fonc.2021.681905)
Supplement: Supplementary file 1 [file Table_1.docx]

**Supplementary Table 1.** Univariate analysis for factors associated with pCR in the luminal subtype.

| **Characteristics** | **Non-pCR**  **(n=306)** | **pCR**  **(n=26)** | ***p*-value** |
| --- | --- | --- | --- |
| **Age (y)**  <50  ≥50 | 177 (57.8%)  129 (42.2%) | 16 (61.5%)  10 (38.5%) | 0.714 |
| **Menopause**  Yes  No | 196 (64.1%)  110 (35.9%) | 17 (65.4%)  9 (34.6%) | 0.892 |
| **Chemotherapy cycles**  3  4  5-8 | 9 (2.9%)  281 (91.8%)  16 (5.2%) | 0 (0%)  23 (88.5%)  3 (11.5%) | 0.306 |
| **Histological type**  Ductal  Lobular  Others | 294 (96.1%)  4 (1.3%)  8 (2.6%) | 20 (76.9%)  1 (3.8%)  5 (19.2%) | 0.001 |
| **Tumor size**  T1  T2  T3 | 28 (9.2%)  219 (71.6%)  59 (19.3%) | 9 (34.6%)  16 (61.5%)  1 (3.8%) | 0.001 |
| **Clinical nodal status**  Negative  Positive | 108 (35.3%)  198 (64.7%) | 15 (57.7%)  11 (42.3%) | 0.023 |
| **Histological Grade**  I  II  III | 17 (5.6%)  256 (83.7%)  33 (10.8%) | 7 (26.9%)  18 (69.2%)  1 (3.8%) | 0.002 |
| **Ki67 expression (%)**  <14  ≥14 | 137 (44.8%)  169 (55.2%) | 3 (11.5%)  23 (88.5%) | 0.002 |
| **ALB**  <40  ≥40 | 124 (40.5%)  182 (59.5%) | 12 (46.2%)  14 (53.8%) | 0.575 |
| **ALP**  <100  ≥100 | 279 (91.2%)  27 (8.8%) | 24 (92.3%)  2 (7.7%) | 0.845 |
| **AAPR**  <0.583  ≥0.583 | 139 (45.4%)  167 (54.6%) | 7 (26.9%)  19 (73.1%) | 0.068 |

ALB, albumin; ALP, alkaline phosphatase; AAPR, albumin-to-alkaline phosphatase ratio; pCR, pathologic complete response.

**Supplementary Table 2.** Multivariate analysis for factors associated with pCR in the luminal subtype.

| **Characteristics** | **Crude OR**  **(95%CI)** | ***p*-value** | **Adjusted OR**  **(95%CI)** | ***p*-value** |
| --- | --- | --- | --- | --- |
| **Histological type**  Ductal  Lobular  Others | Reference  3.675(0.392-34.439)  9.187(2.752-30.677) | 0.254  <0.001 | Reference  2.319(0.058-92.593)  1.297(0.152-11.100) | 0.655  0.812 |
| **Tumor size**  T1  T2  T3 | Reference  0.227(0.092-0.563)  0.053(0.006-0.437) | 0.001  0.006 | Reference  0.092(0.028-0.298)  0.015(0.001-0.172) | <0.001  0.001 |
| **Clinical nodal status**  Negative  Positive | Reference  0.400(0.177-0.901) | 0.027 | Reference  0.552(0.206-1.481) | 0.238 |
| **Histological grade**  I  II  III | Reference  0.171(0.063-0.465)  0.074(0.008-0.648) | 0.001  0.019 | Reference  0.073(0.012-0.451)  0.029(0.002-0.479) | 0.005  0.013 |
| **Ki67 expression (%)**  <14  ≥14 | Reference  6.215(1.827-21.137) | 0.003 | Reference  13.500(3.321-54.882) | <0.001 |
| **AAPR**  <0.583  ≥0.583 | Reference  2.259(0.923-5.531) | 0.074 | Reference  3.245(1.055-9.980) | 0.040 |

AAPR, albumin-to-alkaline phosphatase ratio; pCR, pathologic complete response.

**Supplementary Table 3.** Univariate analysis for factors associated with pCR in the luminal/HER2 subtype.

| **Characteristics** | **Non-pCR**  **(n=154)** | **pCR**  **(n=21)** | ***p*-value** |
| --- | --- | --- | --- |
| **Age (y)**  ＜50  ≥50 | 96 (62.3%)  58 (37.7%) | 10 (47.6%)  11 (52.4%) | 0.195 |
| **Menopause**  Yes  No | 98 (63.6%)  56 (36.4%) | 12 (57.1%)  9 (42.9%) | 0.563 |
| **Chemotherapy cycles**  3  4  5-8 | 2 (1.3%)  140 (90.9%)  12 (7.8%) | 1 (4.8%)  19 (90.5%)  1 (4.8%) | 0.456 |
| **Histological type**  Ductal  Lobular  Others | 154 (100%)  0 (0%)  0 (0%) | 20 (95.2%)  0 (0%)  1 (4.8%) | 0.120 |
| **Tumor size**  T1  T2  T3 | 17 (11.0%)  100 (64.9%)  37 (24.0%) | 5 (23.8%)  13 (61.9%)  3 (14.3%) | 0.206 |
| **Clinical nodal status**  Negative  Positive | 48 (31.2%)  106 (68.8%) | 14 (66.7%)  7 (33.3%) | 0.001 |
| **Histological Grade**  I  II  III | 8 (5.2%)  124 (80.5%)  22 (14.3%) | 4 (19.0%)  14 (66.7%)  3 (14.3%) | 0.060 |
| **Ki67 expression (%)**  <14  ≥14 | 50 (32.5%)  104 (67.5%) | 2 (9.5%)  19 (90.5%) | 0.031 |
| **ALB**  ＜40  ≥40 | 58 (37.7%)  96 (62.3%) | 7 (33.3%)  14 (66.7%) | 0.700 |
| **ALP**  ＜100  ≥100 | 143 (92.9%)  11 (7.1%) | 21 (100%)  0 (0%) | 0.365 |
| **AAPR**  <0.583  ≥0.583 | 58 (37.7%)  96 (62.3%) | 5 (23.8%)  16 (76.2%) | 0.215 |

ALB, albumin; ALP, alkaline phosphatase; AAPR, albumin-to-alkaline phosphatase ratio; pCR, pathologic complete response.

**Supplementary Table 4.** Multivariate analysis for factors associated with pCR in the luminal /HER2 subtype.

| **Characteristics** | **Crude OR**  **(95%CI)** | ***p*-value** | **Adjusted OR**  **(95%CI)** | ***p*-value** |
| --- | --- | --- | --- | --- |
| **Clinical nodal status**  Negative  Positive | Reference  0.226(0.086-0.597) | 0.003 | Reference  0.227(0.083-0.622) | 0.004 |
| **Histological grade**  I  II  III | Reference  0.226(0.060-0.846)  0.273(0.050-1.495) | 0.027  0.135 | Reference  0.176(0.039-0.799)  0.255(0.038-1.696) | 0.024  0.158 |
| **Ki67 expression (%)**  <14  ≥14 | Reference  4.567(1.024-20.378) | 0.047 | Reference  5.038(1.068-23.765) | 0.041 |

pCR, pathologic complete response.

**Supplementary Table 5.** Univariate analysis for factors associated with pCR in the HER2 enriched subtype.

| **Characteristics** | **Non-pCR**  **(n=125)** | **pCR**  **(n=26)** | ***p*-value** |
| --- | --- | --- | --- |
| **Age (y)**  ＜50  ≥50 | 49 (39.2%)  76 (60.8%) | 8 (30.8%)  18 (69.2%) | 0.420 |
| **Menopause**  Yes  No | 57 (45.6%)  68 (54.4%) | 10 (38.5%)  16 (61.5%) | 0.525 |
| **Chemotherapy cycles**  3  4  5-8 | 5 (4.0%)  108 (86.4%)  12 (9.6%) | 0 (0%)  23 (88.5%)  3 (11.5%) | 0.678 |
| **Histological type**  Ductal  Lobular  Others | 122 (97.6%)  2 (1.6%)  1 (0.8%) | 25 (96.2%)  0 (0%)  1 (3.8%) | 0.534 |
| **Tumor size**  T1  T2  T3 | 11 (8.8%)  86 (68.8%)  28 (22.4%) | 2 (7.7%)  19 (73.1%)  5 (19.2%) | 0.939 |
| **Clinical nodal status**  Negative  Positive | 47 (37.6%)  78 (62.4%) | 16 (61.5%)  10 (38.5%) | 0.024 |
| **Histological Grade**  I  II  III | 3 (2.4%)  80 (64.0%)  42 (33.6%) | 3 (11.5%)  13 (50.0%)  10 (38.5%) | 0.077 |
| **Ki67 expression (%)**  <14  ≥14 | 26 (20.8%)  99 (79.2%) | 6 (23.1%)  20 (76.9%) | 0.796 |
| **ALB**  ＜40  ≥40 | 47 (37.6%)  78 (62.4%) | 9 (34.6%)  17 (65.4%) | 0.774 |
| **ALP**  ＜100  ≥100 | 108 (86.4%)  17 (13.6%) | 21 (80.8%)  5 (19.2%) | 0.540 |
| **AAPR**  <0.583  ≥0.583 | 65 (52.0%)  60 (48.0%) | 13 (50.0%)  13 (50.0%) | 0.853 |

ALB, albumin; ALP, alkaline phosphatase; AAPR, albumin-to-alkaline phosphatase ratio; pCR, pathologic complete response.

**Supplementary Table 6.** Multivariate analysis for factors associated with pCR in the HER2 enriched subtype.

| **Characteristics** | **Crude OR**  **(95%CI)** | ***p*-value** | **Adjusted OR**  **(95%CI)** | ***p*-value** |
| --- | --- | --- | --- | --- |
| **Clinical nodal status**  Negative  Positive | Reference  0.377(0.158-0.898) | 0.028 | Reference  0.311(0.124-0.784) | 0.013 |
| **Histological grade**  I  II  III | Reference  0.163(0.030-0.893)  0.238(0.042-1.360) | 0.037  0.107 | Reference  0.105(0.017-0.633)  0.157(0.025-0.983) | 0.014  0.048 |

pCR, pathologic complete response.

**Supplementary Table 7.** Univariate analysis for factors associated with pCR in the TNBC subtype.

| **Characteristics** | **Non-pCR**  **(n=92)** | **pCR**  **(n=30)** | ***p*-value** |
| --- | --- | --- | --- |
| **Age (y)**  ＜50  ≥50 | 53 (57.6%)  39 (42.4%) | 20 (66.7%)  10 (33.3%) | 0.379 |
| **Menopause**  Yes  No | 57 (62.0%)  35 (38.0%) | 21 (70.0%)  9 (30.0%) | 0.426 |
| **Chemotherapy cycles**  3  4  5-8 | 4 (4.3%)  74 (80.4%)  14 (15.2%) | 1 (3.3%)  27 (90.0%)  2 (6.7%) | 0.556 |
| **Histological type**  Ductal  Lobular  Others | 84 (91.3%)  3 (3.3%)  5 (5.4%) | 30 (100%)  0 (0%)  0 (0%) | 0.328 |
| **Tumor size**  T1  T2  T3 | 5 (5.4%)  66 (71.7%)  21 (22.8%) | 6 (20.0%)  19 (63.3%)  5 (16.7%) | 0.068 |
| **Clinical nodal status**  Negative  Positive  **Histological Grade**  I  II  III | 45 (48.9%)  47 (51.1%)  6 (6.5%)  53 (57.6%)  33 (35.9%) | 26 (86.7%)  4 (13.3%)  2 (6.7%)  17 (56.7%)  11 (36.7%) | <0.001  1.000 |
| **Ki67 expression (%)**  <14  ≥14 | 15 (16.3%)  77 (83.7%) | 2 (6.7%)  28 (93.3%) | 0.237 |
| **ALB**  ＜40  ≥40 | 37 (40.2%)  55 (59.8%) | 17 (56.7%)  13 (43.3%) | 0.115 |
| **ALP**  ＜100  ≥100 | 83 (90.2%)  9 (9.8%) | 28 (93.3%)  2 (6.7%) | 0.605 |
| **AAPR**  <0.583  ≥0.583 | 44 (47.8%)  48 (52.2%) | 8 (26.7%)  22 (73.3%) | 0.042 |

ALB, albumin; ALP, alkaline phosphatase; AAPR, albumin-to-alkaline phosphatase ratio; pCR, pathologic complete response.

**Supplementary Table 8.** Multivariate analysis for factors associated with pCR in the TNBC subtype.

| **Characteristics** | **Crude OR**  **(95%CI)** | ***p*-value** | **Adjusted OR**  **(95%CI)** | ***p*-value** |
| --- | --- | --- | --- | --- |
| **Tumor size**  T1  T2  T3 | Reference  0.240(0.068-0.873)  0.198(0.043-0.922) | 0.030  0.039 | Reference  0.193(0.044-0.839)  0.212(0.038-1.201) | 0.028  0.080 |
| **Clinical nodal status**  Negative  Positive | Reference  0.147(0.048-0.456) | 0.001 | Reference  0.153(0.047-0.494) | 0.002 |
| **AAPR**  <0.583  ≥0.583 | Reference  2.521(1.018-6.242) | 0.046 | Reference  2.868(1.048-7.849) | 0.040 |

AAPR, albumin-to-alkaline phosphatase ratio; pCR, pathologic complete response.
